# Relative Roles of Listeriolysin O, InlA, and InlB in Listeria monocytogenes Uptake by Host Cells

**DOI:** 10.1128/IAI.00555-18

**Published:** 2018-09-21

**Authors:** Christopher C. Phelps, Stephen Vadia, Eusondia Arnett, Yubo Tan, Xiaoli Zhang, Sarika Pathak-Sharma, Mikhail A. Gavrilin, Stephanie Seveau

**Affiliations:** a Department of Microbial Infection and Immunity, The Ohio State University, Columbus, Ohio, USA; b Department of Microbiology, The Ohio State University, Columbus, Ohio, USA; c Department of Biomedical Informatics, Center for Biostatistics, The Ohio State University, Columbus, Ohio, USA; d Pulmonary, Allergy, Critical Care and Sleep Medicine, Davis Heart and Lung Research Institute, Department of Internal Medicine, The Ohio State University, Columbus, Ohio, USA; e Infectious Disease Institute, The Ohio State University, Columbus, Ohio, USA; University of Illinois at Chicago

**Keywords:** InlA, InlB, internalin, Listeria monocytogenes, listeriolysin O, listeriosis, host cell invasion, pore-forming toxins

## Abstract

Listeria monocytogenes is a facultative intracellular pathogen that infects a wide variety of cells, causing the life-threatening disease listeriosis. L. monocytogenes virulence factors include two surface invasins, InlA and InlB, known to promote bacterial uptake by host cells, and the secreted pore-forming toxin listeriolysin O (LLO), which disrupts the phagosome to allow bacterial proliferation in the cytosol. In addition, plasma membrane perforation by LLO has been shown to facilitate L. monocytogenes internalization into epithelial cells. In this work, we tested the host cell range and importance of LLO-mediated L. monocytogenes internalization relative to the canonical invasins, InlA and InlB. We measured the efficiencies of L. monocytogenes association with and internalization into several human cell types (hepatocytes, cytotrophoblasts, and endothelial cells) using wild-type bacteria and isogenic single, double, and triple deletion mutants for the genes encoding InlA, InlB and LLO. No role for InlB was detected in any tested cells unless the InlB expression level was substantially enhanced, which was achieved by introducing a mutation (prfA*) in the gene encoding the transcription factor PrfA. In contrast, InlA and LLO were the most critical invasion factors, although they act in a different manner and in a cell-type-dependent fashion. As expected, InlA facilitates both bacterial attachment and internalization in cells that express its receptor, E-cadherin. LLO promotes L. monocytogenes internalization into hepatocytes, but not into cytotrophoblasts and endothelial cells. Finally, LLO and InlA cooperate to increase the efficiency of host cell invasion by L. monocytogenes.

## Abstract (precis)

Listeria monocytogenes is a facultative intracellular pathogen that infects a wide variety of cells, causing the life-threatening disease listeriosis. L. monocytogenes virulence factors include two surface invasins, InlA and InlB, known to promote bacterial uptake by host cells, and the secreted pore-forming toxin listeriolysin O (LLO), which disrupts the phagosome to allow bacterial proliferation in the cytosol.

## INTRODUCTION

Listeria monocytogenes is a Gram-positive, facultative intracellular bacterium responsible for the foodborne disease listeriosis. Listeriosis is a life-threatening condition for elderly and immunocompromised individuals (1). In these populations, the bacterium can propagate from the intestines to the blood and further disseminate, causing septicemia and meningoencephalitis (1–3, 6). During pregnancy, susceptibility to L. monocytogenes infection is drastically increased and the bacterium can cross the placental barrier, leading to spontaneous abortion, preterm labor, stillbirth, and severe infections of the newborn (1a–1c). An important virulence attribute of L. monocytogenes is its ability to infect numerous cell types, from macrophages to normally nonphagocytic cells such as intestinal and placental epithelial cells, endothelial cells, and neurons (1). The wide host cell range of this pathogen is thought to be critical for crossing the tightest barriers of the human host, i.e., the placental and blood-brain barriers.

The expression of major virulence factors that mediate the L. monocytogenes intracellular life cycle is controlled by PrfA (8–10), which activates transcription in response to a variety of environmental signals, including temperature (11) and nutrient availability (12–14). Two of these virulence factors are the surface proteins InlA and InlB, depicted as the major invasins responsible for L. monocytogenes uptake by normally nonphagocytic cells (4, 15, 16). InlA (internalin) is covalently anchored to the peptidoglycan through its C-terminal LPXTG motif (16, 17), whereas InlB is retained noncovalently at the cell surface via electrostatic interaction between three C-terminal glycine and tryptophan (GW) repeat domains and lipoteichoic acids of the bacterial cell wall (18). The adherens junction protein E-cadherin has been identified as the sole InlA receptor (19), and several host surface proteins, c-Met (or HGF receptor) (20), gC1Q receptor (21), and surface glycosaminoglycans (22), have been identified as InlB receptors. The N-terminal leucine-rich repeat (LRR) domain of InlB binds to c-Met, whereas its C-terminal moiety binds to glycosaminoglycans and gC1Q receptor in addition to being the lipoteichoic acid anchor (21, 22). InlA mediates bacterial entry only into cells expressing E-cadherin, whereas InlB is a more versatile invasin, as its receptors are widely expressed. Importantly, InlA and InlB are species specific: humans and gerbils are permissive to both InlA and InlB, while rabbits/guinea pigs and mice are permissive only to InlA and InlB, respectively (24). It has been proposed that InlB acts as a facilitator of the InlA-dependent invasion pathway in enterocytes (25, 26) and that InlA and InlB, but not listeriolysin O (LLO), are the two most important invasion factors for crossing the intestinal barrier (6, 25, 26).

Upon ingestion by host cells, L. monocytogenes is confined within a vacuole or phagosome that is disrupted by the secreted pore-forming toxin LLO and phospholipases to release the bacterium into the cytosol, where it divides and from which it infects other cells by cell-to-cell spreading (27–30). The role of LLO in mediating vacuolar escape is certainly a major role of this toxin, as the absence of LLO leads to a marked deficiency in intracellular replication of phagocytosed bacteria (30). The role of LLO was considered to be specifically restricted to the disruption of the phagosome (31), but additional roles have been attributed to this toxin. In particular, it has been shown that LLO, secreted by extracellular bacteria, perforates the host cell plasma membrane during the early stage of infection; therefore, LLO secretion and membrane perforation precede the formation of the phagosome (32, 35). Perforation of the host cell plasma membrane activates several signaling pathways (28). One outcome of LLO-induced signaling is the internalization of L. monocytogenes into epithelial cell lines (HepG2, HeLa, and Hep2 cells) (33–35) and professional phagocytes (human neutrophils and murine bone marrow-derived macrophages) (36). However, once bacteria are opsonized, the contribution of LLO in bacterial uptake by professional phagocytes becomes negligible. In addition, LLO-mediated plasma membrane perforation by cytosolic bacteria was recently proposed to facilitate cell-to-cell spreading (37).

Because InlA and InlB are described as the most important factors controlling L. monocytogenes uptake by normally nonphagocytic cells, it was necessary to establish whether the role of LLO is significant in comparison to these two canonical invasins. It was also necessary to determine if LLO plays a general role in inducing L. monocytogenes internalization in all cell types. To address these questions, we used human hepatocytes and cytotrophoblasts, because they are known to be infected by L. monocytogenes during listeriosis (1). It is also known that L. monocytogenes can infect endothelial cells *in vitro* and may infect these cells *in vivo* to cross the blood-brain and placental-fetal barriers (38–43). As such, endothelial cells were included in this work. Although enterocytes that make up the intestinal barrier are of critical importance for the establishment of listeriosis, previous work has convincingly shown that crossing the intestinal barrier is InlA dependent and LLO independent, so enterocytes were not included (6). To quantify and compare the roles of the three invasins, we used a fluorescence-based microscopy assay that directly measures the efficiency of bacterial association with host cells and the efficiency of their internalization.

## RESULTS

### LLO, InlA, and InlB expression levels in single and double deletion mutants.

**FIG 1:**
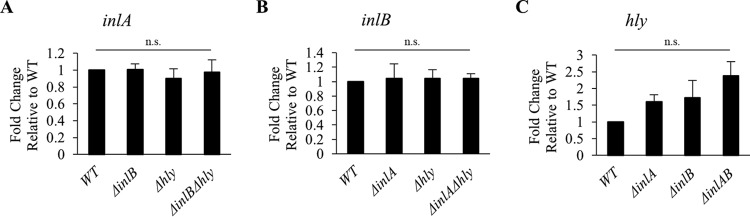
*inlA*, *inlB*, and *hly* mRNA quantification. Reverse transcription quantitative real-time PCR (RT-qPCR) was performed to measure *inlA*, *inlB*, *hly*, *gap*, and *rpoB* transcripts from L. monocytogenes WT and isogenic deletion mutants. The housekeeping genes, *gap* and *rpoB*, were used to normalize the expression of *inlA* (A), *inlB* (B), and *hly* (C). Results are the average fold change in gene expression ± standard error of the mean (SEM) relative to the WT (*n* ≥ 3). Statistical differences from the WT are indicated (n.s., non-statistically significant).

**FIG 2:**
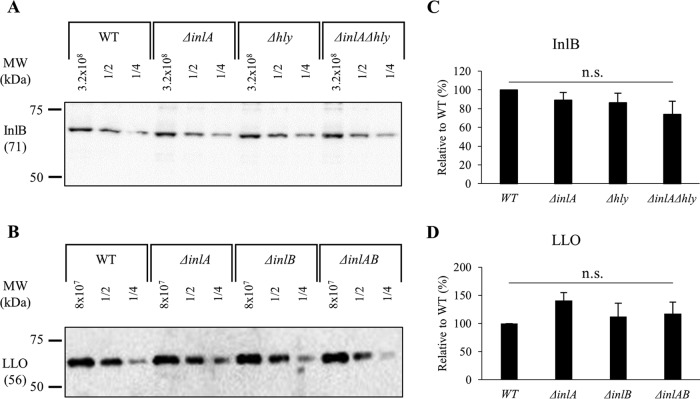
InlB and LLO protein levels. (A and B) L. monocytogenes cell lysates, undiluted and at dilutions of 1/2 and 1/4, were subjected to Western blot analysis using anti-InlB and anti-LLO antibodies. (C and D) Densitometry analysis was performed using ImageJ software. Representative Western blots are shown. Results are the mean ± SEM relative to the WT (*n* ≥ 3). Statistical differences from the WT using data prior to normalization are indicated (n.s., non-statistically significant).

To ensure that deletion of the virulence genes *hly*, *inlA*, and *inlB*, in the single and double deletion L. monocytogenes 10403S mutants, does not affect the expression of the others, the levels of mRNA and proteins of the three invasion factors were measured. Bacteria were grown under the same experimental conditions as for the cell invasion assay and reverse transcription quantitative real-time PCR (RT-qPCR) was used to measure *hly*, *inlA*, and *inlB* mRNA levels. As expected, deletion of one or two virulence genes does not significantly affect the expression of the other genes in comparison to the wild-type (WT) strain (Fig. 1). We then measured the protein expression levels by Western blotting, which required antibodies against LLO, InlB, and InlA. Anti-LLO antibodies are commercially available, but not anti-InlA and anti-InlB. Therefore, we cloned *inlA* and *inlB* genes (without the signal peptide-encoding sequence) into an expression vector (pET29b), purified the recombinant proteins, and obtained purified polyclonal rabbit anti-InlB and -InlA. The anti-InlB antibodies could efficiently detect InlB (see Fig. S1 in the supplemental material), but we were not successful with the anti-InlA antibodies. We then measured LLO and InlB protein expression levels by Western blotting. For rigorous evaluation, we analyzed serial dilutions of cell lysates and performed densitometry analyses of the corresponding bands. As expected, single and double deletions of the *inlA*, *inlB*, or *hly* genes do not significantly affect the expression levels of LLO or InlB (Fig. 2).

### InlA and LLO, but not InlB, control L. monocytogenes uptake by human hepatocytes.

**FIG 3:**
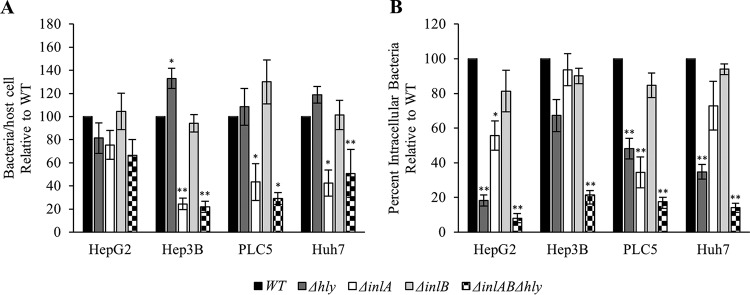
Relative roles of LLO, InlA, and InlB in L. monocytogenes invasion of human hepatocytes. HepG2, Hep3B, PLC5, and Huh7 cells were infected with WT, InlA-deficient (Δ*inlA*), InlB-deficient (Δ*inlB*), LLO-deficient (Δ*hly*), or InlAB- and LLO-deficient (Δ*inlAB Δhly*) bacteria (MOI of 20) for 30 min at 37°C. Cells were washed, fixed, and labeled with fluorescent antibodies and DAPI. (A) The bacterial association efficiency was calculated as the total number of bacteria associated per host cell. The average bacterial association values for the WT strain before normalization were as follows: HepG2, 0.14; Hep3B, 3.13; PLC5, 1.34; Huh7, 0.77. (B) The bacterial internalization efficiency was calculated as the percentage of intracellular bacteria. The average percentages of internalization for the WT strain before normalization were as follows: HepG2, 26.45%; Hep3B, 38.77%; PLC5, 18.29%; Huh7, 33.12%. The minimum numbers of host cells counted were as follows: HepG2, 1,000; Hep3B, 150; PLC5, 600; Huh7, 2,000. The average numbers of WT bacteria counted per experiment were as follows: HepG2, 600; Hep3B, 4,000; PLC5, 2,000; Huh7, 3,000 (with a minimum count of 100 bacteria being required for any mutant with reduced association efficiency). Results are expressed as the mean ± SEM relative to the WT (*n* ≥ 3). Statistical analyses compared each deletion strain to the WT strain and were performed on raw data before normalization (*, *P* < 0.01, **, *P* < 0.001).

To establish the relative roles of the three virulence factors in L. monocytogenes uptake by human hepatocytes, we used four human hepatocyte cell lines (HepG2, Hep3B, PLC5, and Huh7) to rule out any cell line-specific phenotype and draw conclusions that can generally apply to hepatocytes. Hepatocytes were incubated with L. monocytogenes (WT or Δ*hly*, Δ*inlA*, Δ*inlB*, Δ*inlAB*, Δ*inlB* Δ*hly*, Δ*inlA* Δ*hly*, or Δ*inlAB* Δ*hly* mutants) for 30 min at 37°C and were processed for fluorescence microscopy analysis. Full data sets, including association and internalization efficiencies of the eight bacterial strains into the four cell lines, are presented in Fig. S2. We first focused on analyzing data obtained with the single and triple deletion mutants in comparison to WT L. monocytogenes (Fig. 3). Data show that LLO does not promote L. monocytogenes association with hepatocytes. In one of the hepatocyte cell lines (Hep3B), LLO even significantly decreases bacterial association. In contrast, InlA is the only factor that promotes bacterial association with hepatocytes, in three out of the four cell lines. The decreases in association of the *inlA* single deletion mutant and the triple deletion mutant were similar in all hepatocyte cell lines, confirming that among the three factors, InlA is the only adhesin. LLO and InlA, but not InlB, promote internalization of L. monocytogenes, although the role of LLO was more prominent in that function than the role of InlA. In one cell line (Hep3B), single deletion mutants had no internalization phenotype, whereas the triple (Δ*inlAB* Δ*hly*) and double (Δ*inlA* Δ*hly*) (Fig. S2) deletion mutants displayed a significant decrease in internalization. The latter result shows that LLO and InlA can exert a redundant role in L. monocytogenes internalization. To our surprise, no role for InlB was detected in L. monocytogenes association and internalization into the four hepatocyte cell lines when single, double, and triple deletion mutants were considered (Fig. 3 and Fig. S2). This prompted us to clarify this result.

### InlB-mediated L. monocytogenes internalization is dependent on InlB expression level.

**FIG 4:**
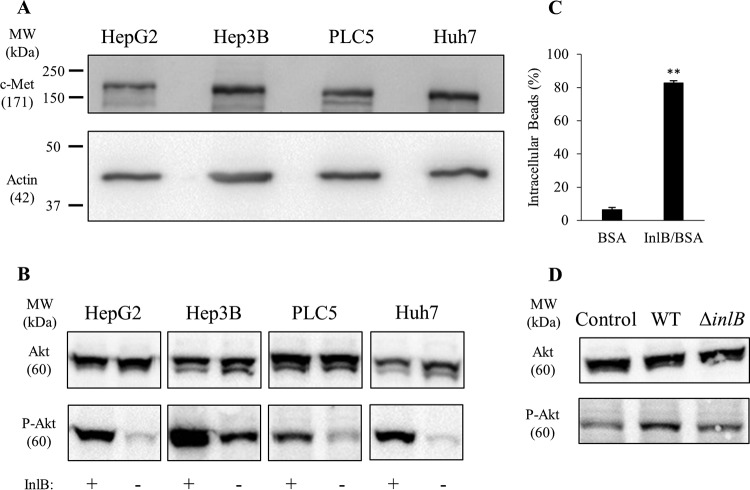
The InlB/c-Met signaling pathway is functional in hepatocytes. (A) HepG2, Hep3B, PLC5, and Huh7 cell lysates were subjected to Western blot analysis using anti-c-Met and anti-actin (loading control) antibodies. (B) Cells were exposed, or not, to 1.25 nM InlB for 5 min, and cell lysates were subjected to Western blot analysis using anti-Akt and anti-phospho-Akt antibodies. A representative Western blot is presented (*n* = 3). (C) HepG2 cells were incubated with BSA- or BSA/InlB-coated beads for 30 min at 37°C (MOI of 5). Results are expressed as the average percentage of internalization ± SEM (*n* = 4; *, *P* < 0.01; **, *P* < 0.001). (D) After infection with WT or Δ*inlB* bacteria (MOI of 20) for 30 min, HepG2 cells were lysed and lysates were subjected to Western blot analysis using anti-Akt and anti-phospho-Akt antibodies. A representative Western blot is shown (*n* = 3). MW, molecular weight.

**FIG 5:**
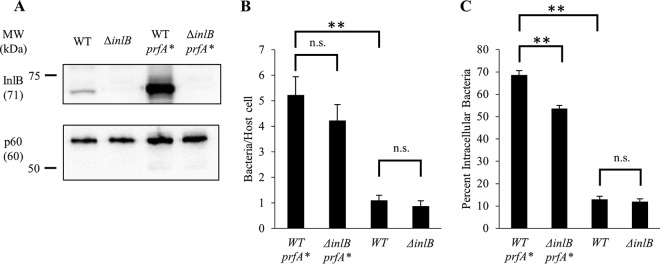
A prfA* mutation in L. monocytogenes strain 10403S leads to increased production of InlB and InlB-dependent hepatocyte invasion. (A) Bacterial lysates (1.6 × 10^8^ cells) were subjected to Western blot analysis using anti-InlB and anti-p60 (loading control) antibodies. A representative Western blot is shown (*n* = 3). (B and C) PLC5 cells were infected with WT, Δ*inlB*, WT-prfA*, or ΔinlB-prfA* bacteria (MOI of 5) for 30 min at 37°C. Cells were washed, fixed, and labeled with fluorescent antibodies and DAPI. (B) The bacterial association efficiency was calculated as the total number of bacteria associated per host cell. (C) The bacterial internalization efficiency was calculated as the percentage of intracellular bacteria. (B and C) A minimum of 2,000 bacteria were counted per condition, and a minimum of 500 host cells were counted per condition. Results are expressed as the mean ± SEM (*n* = 4; *, *P* < 0.01; **, *P* < 0.001, n.s., non-statistically significant).

The absence of a role for InlB led us to verify that its receptor, c-Met, was expressed and functional in the hepatocyte cell lines used in these studies. As expected, c-Met was expressed in all tested hepatocyte cell lines (Fig. 4A). Previous studies established that InlB activates c-Met-dependent Akt phosphorylation and F-actin remodeling (44–46). As expected, cell exposure to recombinant InB induced a significant increase in Akt phosphorylation in all cell lines (Fig. 4B). As a second approach, live cell imaging showed that hepatocytes exposed to InlB formed dynamic membrane ruffles, which were not observed in the absence of InlB (see Movies S1 to S4 in the supplemental material). Finally, to evaluate if hepatocytes could undergo InlB-dependent phagocytic uptake, we exposed cells to polystyrene beads (1-μm diameter) that were covalently coated with saturating concentrations of InlB or bovine serum albumin (BSA), used as negative control. As shown in Fig. 4C, 80% of InlB-coated beads were internalized by hepatocytes. We then established if InlB produced by L. monocytogenes could stimulate c-Met. HepG2 cells were incubated with WT and Δ*inlB* 10403S strains for 30 min at a multiplicity of infection (MOI) of 20, as performed in the invasion assays. As shown in Fig. 4D, WT but not InlB-deficient bacteria induced Akt phosphorylation. Together, these results demonstrate that the hepatocyte cell lines express a functional c-Met and that InlB from 10403S is expressed in a sufficient amount to activate c-Met signaling. However, InlB produced by 10403S failed to induce significant bacterial entry. We then tested the hypothesis that InlB was not produced in sufficient amounts by strain 10403S to promote bacterial uptake. This hypothesis was based on the fact that the bead surface was coated with a saturating amount of recombinant InlB and the fact that laboratory strains used to show a role for InlB in bacterial internalization express high levels of InlB (15, 18, 20, 39, 40, 44, 47–51). Indeed, the commonly studied laboratory strain EGD expresses a constitutively active variant of the transcriptional regulatory factor PrfA, known as a PrfA* (G145S) variant, which is responsible for high production levels of InlB and other PrfA-regulated virulence factors (14, 47, 52–57). To test if an increase in InlB production in the 10403S background would result in InlB-mediated internalization of L. monocytogenes, we generated prfA* and ΔinlB prfA* strains in the 10403S background by phage transduction (47, 57, 58). We compared the production of InlB between 10403S WT and prfA* strains and report a marked increase in InlB production, as expected (47) (Fig. 5A). The replacement of WT *prfA* with prfA* led to a 5-fold increase in bacterial association (Fig. 5B) and a 7-fold increase in bacterial entry into host cells (Fig. 5C). A comparison of prfA* and ΔinlB prfA* strains showed that, in the prfA* background, InlB plays a significant role in bacterial entry (Fig. 5C), while a comparison of the WT and Δ*inlB* strains shows no difference in either bacterial association or bacterial entry (Fig. 5B and C). Collectively, these data show that a bacterial strain such as 10304S produces enough InlB to activate c-Met, but this amount is not sufficient to affect L. monocytogenes internalization.

### Only InlA, not InlB or LLO, controls L. monocytogenes uptake by human cytotrophoblasts.

**FIG 6:**
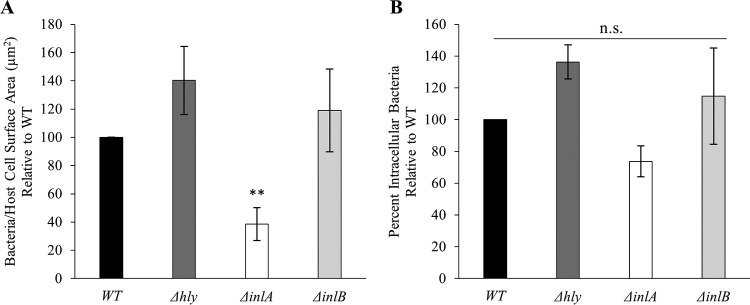
Role of LLO, InlA, and InlB in L. monocytogenes invasion of human cytotrophoblasts. BeWo cells were infected with WT, LLO-deficient (Δ*hly*), InlA-deficient (Δ*inlA*), or InlB-deficient (Δ*inlB*) bacteria (10^6^ bacteria/well) for 30 min at 37°C. The cells were washed, fixed, and labeled with fluorescent antibodies and DAPI. (A) The bacterial association efficiency was calculated as the number of cell-associated bacteria per unit surface area (μm^2^). The average association for the WT strain before normalization was 0.0015 bacteria/μm^2^. (B) The bacterial internalization efficiency was measured as the percentage of intracellular bacteria. The average internalization for the WT strain before normalization was 13.82%. The average number of WT bacteria counted per experimental condition was 5,000, with a minimum count of 100 bacteria being required for any mutant with reduced association efficiency. Results are expressed as the mean ± SEM relative to the WT (*n* ≥ 3). Statistical analyses compared each strain to the WT strain and were performed on raw data before normalization (*, *P* < 0.01; **, *P* < 0.001; n.s., nonsignificant).

We next determined the role of LLO, InlA, and InlB in L. monocytogenes uptake by human cytotrophoblast-like BeWo cells. Cytotrophoblasts are cells of fetal origin located at the interface between maternal and fetal tissues. Invasion of the placenta requires traversal of the cytotrophoblast barrier. No role for LLO in L. monocytogenes association and entry was detected in BeWo cells. Two other cytotrophoblast-like cells, Jeg-3 and JAR, were also tested, leading to the same conclusion (data not shown). Only InlA plays a major role in L. monocytogenes association with BeWo cells, but it does not affect the efficiency of internalization (Fig. 6). Finally, no role for InlB was observed in the invasion of BeWo cells, as previously reported by others using the same bacterial strain (59).

### Uptake of L. monocytogenes by HUVECs is independent of the three invasion factors.

**FIG 7:**
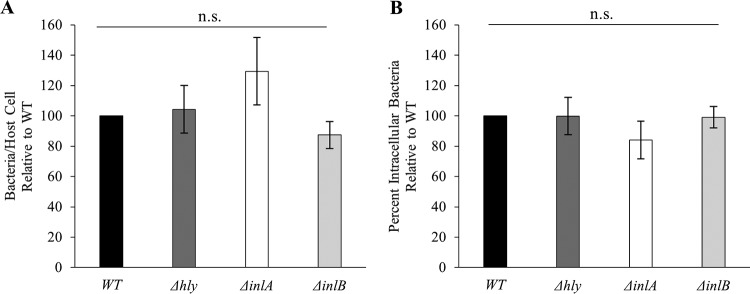
Absence of a role for LLO, InlA, and InlB in L. monocytogenes invasion of human endothelial cells. HUVECs were infected with WT, LLO-deficient (Δ*hly*), InlA-deficient (Δ*inlA*), or InlB-deficient (Δ*inlB*) bacteria (MOI of 5) for 30 min at 37°C. Cells were washed, fixed, and labeled with fluorescent antibodies and DAPI. (A) The bacterial association efficiency was calculated as the number of cell-associated bacteria per human cell. The average association for the WT strain was 0.13 bacteria/host cell. (B) The bacterial internalization efficiency was measured as the percentage of intracellular bacteria. The average internalization efficiency for the WT strain was 13.23%. The average number of WT bacteria counted per experiment was 500, and a minimum of 2,000 host cells were counted per condition. Results are expressed as the mean ± SEM relative to the WT (*n* ≥ 3). Statistical analyses compared each strain to the WT strain and were performed on raw data before normalization (n.s., nonsignificant).

We next assessed the role of LLO, InlA, and InlB in the uptake of L. monocytogenes by human umbilical vein endothelial cells (HUVECs). We used the low MOI of 5 because HUVECs are severely damaged at higher MOIs due to LLO activity, as we have observed and as recently reported (42). Our data showed no role for InlA, InlB, or LLO in the invasion of HUVECs (Fig. 7). This is congruent with the most recent report in the literature regarding L. monocytogenes strain 10403S and HUVECs that supports the notion that bacterial uptake is largely independent of InlA, InlB, and LLO (42).

### Establishing cooperation between LLO and InlA in L. monocytogenes invasion of hepatocytes.

Hepatocyte infection data indicated an important role for both LLO and InlA in L. monocytogenes host cell invasion. This infection model was therefore appropriate for establishing whether LLO and InlA cooperate to potentiate the efficiency of host cell invasion. The biological expectation for positive cooperation between the two proteins, also referred to as synergism, is that the biological response when both proteins are expressed (when both genes are present) will be greater than the sum of their individual responses (when one of the corresponding genes is deleted) (60). To establish if InlA and LLO display positive cooperation in bacterial association with host cells on entry into host cells, we established four groups: InlA and LLO are both expressed (WT strain), LLO is expressed alone (Δ*inlA* strain), InlA is expressed alone (Δ*hly* strain), and neither of the two proteins is expressed (Δ*inlA* Δ*hly* double deletion mutant). A linear mixed-effects model was used to test this hypothesis: (μ_both_ − μ_neither_) > [(μ_A_ − μ_neither_) + (μ_B_ − μ_neither_)], i.e., μ_both_ − μ_A_ − μ_B_ + μ_neither_ > 0, where both is the WT, neither is the double deletion mutant, and μ is the mean outcome for each group (60). If the *P* value for this test is significant, we claim that there is significant synergistic interaction (positive cooperation) between the two proteins. We used this analytical method to test whether InlA and LLO work synergistically to affect bacterial association and internalization of Listeria monocytogenes. Similar analyses were performed to test for potential positive cooperation between InlB and LLO and between InlB and InlA. Estimates and accompanying statistics are included in Table 1. In the process of bacterial association, no pattern of positive cooperation was observed (Table 1). This is consistent with InlA being the sole contributor to association among the tested invasins. In the process of bacterial internalization, no synergistic effect was observed between InlB and the two other invasins (Fig. S2 and Table 1), confirming that InlB does not affect the uptake of L. monocytogenes (strain 10403S) into human hepatocytes. Only LLO and InlA interact in a synergistic manner to potentiate L. monocytogenes internalization into HepG2 and PLC5 cells.

**TABLE 1:** Invasion factor cooperation analysis^*a*^

Invasion factor combination tested	Cell line	Cooperation in internalization			Cooperation in association		
		Estimate	SE	*P* value	Estimate	SE	*P* value
InlA/LLO	HepG2	13.996	5.9587	**0.0232**	−0.04043	0.0379	0.2917
	Hep3B	−16.8597	9.9539	0.0995	−1.1548	0.6499	0.0848
	PLC5	6.5958	2.9439	**0.0332**	−0.3497	0.4422	0.4357
	Huh7	7.0611	6.8443	0.3114	0.01674	0.2237	0.9409
InlA/InlB	HepG2	7.6389	6.0995	0.2168	−0.02705	0.03911	0.4928
	Hep3B	−2.8172	10.1881	0.7838	−0.4551	0.6875	0.5126
	PLC5	3.645	3.3279	0.2827	−0.6872	0.4973	0.1779
	Huh7	−1.0749	7.8543	0.8922	−1.0749	7.8543	0.8922
InlB/LLO	HepG2	8.3178	5.4892	0.1365	−0.07209	0.03502	**0.0452**
	Hep3B	−3.0958	9.4808	0.746	0.4169	0.6232	0.5082
	PLC5	0.9946	2.9439	0.738	0.04456	0.4422	0.9205
	Huh7	2.6579	7.1474	0.7129	2.6579	7.1474	0.7129

a The estimate is the result of the synergistic interaction tests described in Results. Statistically significant *P* values (<0.05) indicate positive (synergistic) cooperation. SE, standard error. Boldface indicates statistically significant *P* values.

## DISCUSSION

This work focused on establishing the relative roles of LLO, InlA, and InlB in L. monocytogenes (strain 10403S) association with and internalization into normally nonphagocytic human cells. The data show that LLO activity is cell type dependent, as LLO plays a significant role in L. monocytogenes internalization into hepatocytes but not into cytotrophoblasts or endothelial cells. InlA and LLO are the two virulence factors that significantly contribute to the invasion of human hepatocytes, with InlA playing a significant role as an adhesin and LLO as an invasin. To our surprise, no role for InlB was detected unless the *prfA* gene was replaced by a constitutively active prfA* mutant, indicating that higher expression levels of InlB are required for InlB-mediated bacterial internalization.

Studies that identified the L. monocytogenes virulence factors controlling host cell invasion have traditionally used the gentamicin survival assay. This assay robustly measures bacterial intracellular survival but presents some limitations. First, it indiscriminately and collectively reports the efficiencies of bacterial association and internalization. Second, host cell perforation by LLO allows for diffusion of gentamicin and potential targeting of intracellular bacteria (35). Finally, this assay generally involves long incubation times, which can be sufficient for intracellular bacterial division or killing. Because of these limitations, we analyzed cells infected for only 30 min at a low MOI and in the absence of gentamicin, using a fluorescence microscopy approach (61). Microscope automation allows for rapid acquisitions of a high number of images, and software-assisted analytical tools considerably decrease the time for analysis. Importantly, this approach specifically quantifies with sensitivity and accuracy the efficiencies of bacterial attachment and association with host cells (61).

No role for InlB was initially detected in the present work. This result was unexpected, because numerous studies report that InlB promotes host cell invasion (15, 20, 40, 44, 48, 49). Using the hepatocyte model, we showed that the InlB receptor, c-Met, was expressed and functional. In addition, the amount of InlB produced by L. monocytogenes 10403S under our experimental conditions was sufficient to activate c-Met-dependent signaling but not bacterial internalization (Fig. 3 and 4). Furthermore, hepatocytes could massively internalize polystyrene beads coated with high concentrations of recombinant InlB (Fig. 4C). Studies that characterized the role of InlB in host cell invasion mostly used strain EGD, which carries a mutation in the gene coding for the master regulator of the virulence gene *prfA* (designated prfA*), leading to high expression levels of InlB among other virulence factors (47). Among all sequenced L. monocytogenes strains analyzed, the prfA* mutation is very rarely observed (47). When the prfA* mutation was introduced into EGD-e, *inlB* transcription was increased over 40-fold (47). This led us to hypothesize that the strain used in our study, 10403S, may not produce enough InlB for productive bacterial internalization. To test this hypothesis, we replaced the WT *prfA* allele with a prfA* allele in the 10403S background and consequently observed a marked increase in InlB production and a statistically significant role for InlB in bacterial internalization. Together, these data support the idea that the level of expression of InlB is critical for bacterial internalization. Therefore, it is reasonable to extrapolate that any conditions, including different bacterial cell growth conditions or environmental conditions, that substantially increase InlB expression would favor InlB-dependent internalization. For example, the transcription level of *inlB* in strain EGD-e is increased in human blood and the murine intestine (62). One should also consider that the role of InlB observed at later time points of infection may be related to bacterial intracellular survival and/or multiplication and not to bacterial internalization.

As expected, InlA promotes invasion of cells that express its receptor, E-cadherin (19). Importantly, the role of InlA was substantial even in strain 10403S expressing wild-type *prfA*. Few studies have focused on distinguishing the role of E-cadherin in anchoring the bacterium to the host cell surface from its role in stimulating bacterial internalization. It was initially proposed that the InlA–E-cadherin interaction promotes both anchoring and internalization, since the intracellular domain of E-cadherin and its association with the F-actin cytoskeleton were necessary for InlA-dependent L. monocytogenes uptake by fibroblasts (63). More recent work studying L. monocytogenes invasion of MDCK epithelial cells expressing wild-type E-cadherin or the glycosylphosphatidylinositol (GPI)-anchored extracellular domain of E-cadherin concluded that the InlA–E-cadherin interaction anchors the bacterium to the host cell surface but is dispensable for F-actin-dependent internalization of the bacterium (64). Our results are in accordance with both studies. We report that the primary function of the InlA–E-cadherin interaction is to anchor the bacterium to the host surface, but this interaction can also control the efficiency of bacterial internalization in some, but not all, cell lines.

LLO plays a critical role in L. monocytogenes internalization into hepatocytes. Other studies established that the formation of LLO pores on the plasma membrane activates the following signaling cascade: influx of extracellular Ca^2+^, activation of Ca^2+^-dependent conventional protein kinase C upstream from the Rho GTPase Rac1, and Arp2/3-dependent formation of F-actin-rich membrane projections that promote internalization of the bacterium (34, 35, 65). Because LLO targets all membranes that contain cholesterol, it was expected that LLO would activate bacterial internalization in all animal cells, including cytotrophoblasts and HUVECs, but to our surprise, this was not the case. However, hepatocytes are not the only cells thus far identified to undergo LLO-dependent L. monocytogenes internalization, as this was also reported in HeLa cells, Hep2 cells, human neutrophils, and macrophages (34). Furthermore, LLO-dependent internalization has been demonstrated for L. monocytogenes strains 10403S, L028, and EGD (33, 34). The difference in host cell response to LLO should be investigated further to understand what makes some cell types permissive to the LLO-dependent entry pathway. This would be useful for understanding how pathogens can generally take advantage of plasma membrane perforation to gain entry into host cells (66, 67).

We report that InlA and LLO cooperate in an additive or synergistic fashion depending on the cell line. Though the mechanism by which LLO and InlA cooperate is still unknown, two non-mutually exclusive hypotheses can be envisioned. First, by anchoring L. monocytogenes to the host cell, InlA increases local LLO concentration and thereby LLO-dependent internalization. Along this line, InlA likely served as the adhesin and LLO promoted the signaling cascade for bacterial internalization into MDCK cells expressing GPI-anchored E-cadherin (64). Second, LLO- and InlA-induced signaling cascades may potentiate the activation of common transducers for the remodeling of F-actin and bacterial engulfment (65, 68).

Most studies that addressed the roles of InlA, InlB, and LLO utilized the laboratory strain EGD, EGD-e, or 10403S, which all belong to serovar 1/2a. EGD is derived from the strain of L. monocytogenes isolated from guinea pigs in 1926 (69). EGD-e is thought to be a derivative of strain EGD (47, 70). 10403S is a derivative of strain 10403, a strain initially isolated from a human skin lesion (71). Of these strains, EGD-e is the most virulent in mice and has been shown to express high levels of some of the PrfA-stimulated genes despite the absence of the prfA* allele (47). L. monocytogenes strains associated with clinical cases and outbreaks of listeriosis belong predominantly to serovars 1/2a, 1/2b, and 4b, with greater than 50% of isolates belonging to serovar 4b (72, 73). Characterization of virulence factors in clinical strains seems to be lacking. A role for InlA in the invasion of Caco-2 cells has been demonstrated with a clinical isolate (Scott A, serotype 4b) from an outbreak of listeriosis in Massachusetts in 1983 (74, 75). One epidemiological study reported that 96% of clinical isolates, and only 65% of food isolates, express full-length InlA (76), and other studies have similarly found a higher prevalence of full-length InlA in strains associated with human and animal infections, with more strains expressing truncated InlA in food isolates (77–79). Other work has found that LLO- and InlB-encoding genes are highly prevalent in clinical strains (80). However, these studies emphasize the importance of InlA, InlB, and LLO as virulence factors but do not directly inform on their mechanism of action *in vivo. In vivo* studies using animal models also established a role for these three virulence factors. Of the three factors, LLO is the most important for virulence, as LLO-deficient strains are avirulent, so dissecting its role *in vivo* is challenging. In mice infected with 10403S or EGD-e, InlB does not affect liver and spleen colonization or the 50% lethal dose (LD_50_) (81, 82). One recent study infecting E-cadherin-humanized mice and gerbils with EGD (prfA*) showed that neither InlA nor InlB affected infection of the liver (7). The same study also showed that InlA is important for infection of the intestines, colon, and cecum and that both InlA and InlB contribute to infection of the placenta and fetus.

In conclusion, to successfully cross the host barriers and invade multiple tissues, L. monocytogenes uses a collection of virulence factors that collectively facilitate bacterial anchoring to host cells and successive internalization. It appears that InlA is the major adhesin, while InlA, LLO, and InlB can stimulate bacterial internalization alone or in concert with InlA. Collectively, the three factors are conserved among clinical strains, but their roles likely vary in a tissue- and strain-dependent fashion.

## MATERIALS AND METHODS

### Bacterial strains and culture.

Escherichia coli XL1-Blue and BL21(DE3) were grown in Luria-Bertani (LB) broth under agitation at 37°C. Plasmids were maintained with either ampicillin (50 μg/ml) or kanamycin (30 μg/ml), as indicated. Wild-type (WT) L. monocytogenes (EGD-e) was a gift from Pascale Cossart (Pasteur Institute, Paris, France) (Table 2). WT L. monocytogenes (10403S) and Δ*hly* (DP-L2161), Δ*inlA* (DP-L4405), Δ*inlB* (DP-L4406), and Δ*inlAB* (DP-L4404) isogenic mutants were gifts from Daniel Portnoy (UC Berkeley, CA, USA). Strain 10403S, a member of lineage II and serotype 1/2a, is a streptomycin-resistant derivative of strain 10403 (47, 71), which was originally isolated from a human skin lesion in 1968 (83). The Δ*inlAB* Δ*hly* triple deletion mutant was developed previously (34). Δ*inlA* Δ*hly* and Δ*inlB* Δ*hly* double deletion mutants were constructed using DP-L4405 and DP-L4406, respectively, by knocking out the *hly* gene via allelic exchange using the pKSV7 integration shuttle vector and primers listed in Table 3, as described previously (34, 84). The deletion of *hly* was confirmed by PCR using primers listed in Table 3. L. monocytogenes strains were grown overnight under agitation at 37°C in brain heart infusion (BHI) (BD Biosciences). For invasion assays, overnight cultures were diluted 20-fold in BHI and grown at 37°C until an optical density at 600 nm (OD_600_) of 0.7 to 0.8 was reached. Cells were washed three times in sterile, 37°C phosphate-buffered saline (PBS) and diluted to the indicated multiplicity of infection (MOI) in appropriate mammalian cell culture medium without serum or antibiotic.

**TABLE 2:** L. monocytogenes strains used in this study

Strain	Genotype	Source or reference
EGD-e	Wild type	70
10403S	Wild type	87
DP-L2161	10403S Δ*hly*	88
DP-L4405	10403S Δ*inlA*	59
DP-L4406	10403S Δ*inlB*	59
DP-L4404	10403S Δ*inlAB*	59
SL33	10403S Δ*inlA* Δ*hly*	This study
SL40	10403S Δ*inlB* Δ*hly*	This study
SL20	10403S Δ*inlAB* Δ*hly*	This study
NF-L1177	10403S *prfA* G145S *actA-gus-neo-plcB*	89
SL64	DP-L4406 *prfA* G145S *actA-gus-neo-plc*	This study

**TABLE 3:** Primers used in this study^*a*^

Purpose of constructs	Oligonucleotide sequence (5′–3′)	Reference
*C*onstruction of Δ*hly* strains	Forward: GGG AAT TCA ATT GTT GAT ACA ATG ACA TC	88
	Reverse: GGC TGC AGG GTC TTT TTG GCT TGT GTA T	88
Primers to amplify the *hly* ORF	Forward: CCG TCG GAT CCA TGA AAA AAA TAA TGC TAG TTT TTATTACAC	88
	Reverse: ATC CGC GCT GCA GTT CGA TTG GAT TAT CTA CTT TAT TAC	88
pET29b-*inB6His* (bp 106 to 1890)	Forward: AAC GTG CAT ATG GAG ACT ATC ACC GTG CCA ACG	This study
	Reverse: ATT CTC GAG TTT CTG TGC CCT TAA ATT AGC TGC	This study
Sequencing *prfA* mutants	Forward: CTA TCT GTT GCA GCT CTT CTT GG	This study
	Reverse: CAG CTA ACA ATT GTT GTT ACT GCC	
Confirm *gus-neo* insertion (prfA* mutants)	Forward: GCA GTC AAT TAA TAT GCC GAG CC	This study
	Reverse: CGG ACC AAC TAA GTT TAT GTG G	This study
Hydrolysis primers and probes for qPCR for gene target		
*inlA*	Forward: GGC AAA GAA ACA ACC AAA GAA G	This study
	Reverse: GGG CAT CAA ACC AAC CAA	This study
	Probe: AT TGA CTG AAC CAG CTA AGC CCG T	This study
*inlB*	Forward: CCG AGC ACT TAA CAC ATT CTA C	This study
	Reverse: TTA TCT GCT ACC GGG ACT TTA T	This study
	Probe: ATG TCA GCG CCA ATA AAG CTG GC	This study
*hly*	Forward: CTG GTT TAG CTT GGG AAT GG	This study
	Reverse: ATT TCG GAT AAA GCG TGG TG	This study
	Probe: TGA TGA CCG GAA CTT ACC ACT TGT GA	This study
*gap*	Forward: TCA CAG CGC AAG ACA AAG	This study
	Reverse: ACT GTT TCA GTT CCG TCT AAT G	This study
	Probe: TG TTA TCT CCG CTC CAG CAA CTG G	This study
*rpoB*	Forward: TGT AAA ATA TGG ACG GCA TCG T	90
	Reverse: GCT GTT TGA ATC TCA ATT AAG TTT GG	90
	Probe: CT GAT TCG CGC AAA ACT TCT ACG CG	90

a All probes have a 5' 6-FAM reporter dye and a 3' Iowa Black FQ quencher.

### Transduction and prfA* mutant isolation.

U153 bacteriophage (85) was used to infect L. monocytogenes strain NF-L1177 (prfA* G145S *actA-gus-neo-plcB*), and the phages were recovered and used to transduce the prfA* (leading to G145S) *actA-gus-neo-plcB* to the target strains, WT 10403S and the Δ*inlB* mutant, as previously described (U153 bacteriophage and strain NF-L1177 were gifts from Nancy Freitag [University of Illinois, Chicago, IL]) (52, 58). Transductants were selected by plating the mixture of phage and bacteria on BHI/agar plates (5 μg/ml neomycin) for 2 days at 37°C. Neomycin-resistant mutants were further screened by plating on BHI/agar plates containing 5 μg/ml neomycin plus 50 μg/ml 5-bromo-4-chloro-3-indolyl-β-d-glucuronic acid (X-gluc) to confirm the prfA* mutation and the downstream *actA-gus-neo-plcB* transcription fusion. The *actA-gus-neo-plcB* insertion was then confirmed by PCR, and the *prfA* G145S mutation was confirmed by sequencing using primers described in Table 3.

### RNA purification, reverse transcription, and RT-qPCR.

For RNA purification, L. monocytogenes was cultured in BHI under agitation at 37°C to an OD_600_ of 0.7 to 0.8. RNA was purified from 10^9^ bacteria and subsequently treated with RNase-free DNase as described previously (12). RNA concentration and purity were measured via a NanoDrop ND-1000 spectrophotometer. RNA integrity was determined on a 1.2% agarose gel. Reverse transcription was performed using a high-capacity RNA-to-cDNA kit (Applied Biosystems) according to the manufacturer's instructions. Duplicate reaction mixtures lacking the reverse transcriptase enzyme were performed in parallel, and these samples were used in RT-qPCR to test for residual DNA contamination. RT-qPCR was performed using a CFX96 real-time system and a C1000 thermal cycler (Bio-Rad). All reactions were performed in 96-well plates using 1.5 ng of converted cDNA, iQ Supermix (Bio-Rad), forward and reverse oligonucleotide primers, and hydrolysis probes (Table 3). No-reverse-transcriptase (NRT) samples were used as negative controls. *inlA*, *inlB*, and *hly* gene expression was normalized to housekeeping genes *gap* and *rpoB*. Fold changes in gene expression are relative to that of WT L. monocytogenes. Primer and probe concentrations were optimized by testing a concentration gradient of all oligonucleotides as described previously (86). All primer/probe sets yielded reaction efficiencies of ∼100%. All RT-qPCR hydrolysis probes include a 5' 6-FAM reporter dye and a 3' Iowa Black FQ quencher. Samples were analyzed in triplicate by RT-qPCR.

### InlB purification and generation of anti-InlB rabbit polyclonal antibodies.

The *inlB* gene, excluding the signal sequence (bp 106 to 1890), was amplified from genomic DNA of L. monocytogenes strain EGD-e using primers (Table 3) that contain NdeI and XhoI restriction sites. This DNA fragment was ligated into the pET29b expression vector upstream of the C-terminal 6His tag sequence. The resulting expression vector, pET29b-*inlB*, was transformed into Escherichia coli strain BL21(DE3). For expression of recombinant protein, this strain was grown at 37°C until an OD_600_ of 0.6 was reached, and expression of recombinant InlB-6His was induced by the addition of 1 mM IPTG (isopropyl-β-d-thiogalactopyranoside) (48). After 5 h of induction, the bacteria were pelleted and suspended in binding buffer (5 mM imidazole, 500 mM NaCl, and 50 mM HEPES, pH 7.9) and lysed with a French press. The crude lysate was centrifuged, and the supernatant was incubated with Ni-nitrilotriacetic acid (Ni-NTA) agarose (Qiagen). After washes, the protein was eluted and dialyzed overnight. Purified recombinant InlB was sent to GenScript (Piscataway, NJ, USA) to generate rabbit anti-InlB polyclonal antibodies. To immunize rabbits, recombinant InlB and complete Freund's adjuvant were administered via subcutaneous injection. After the primary immunization, three boosts were performed over the course of 66 days. InlB-specific IgG antibodies were purified from serum by affinity chromatography using a Sepharose 4B gel coupled to recombinant InlB. The specificity of the antibodies was ensured by Western blotting of WT and *inlB* deletion mutant L. monocytogenes strains (see Fig. S1 in the supplemental material).

### Mammalian cell culture.

The human hepatocyte cell line HepG2 (HB-8065) was purchased from ATCC. The human hepatocyte cell lines Hep3B (HB-8064; ATCC), PLC5 (CRL-8024; ATCC), and Huh7 (Health Science Research Resources Bank, Osaka, Japan; JCRB0403) were gifts from Ching-Shih Chen (The Ohio State University, OH, USA). HepG2, Hep3B, and PLC5 cells were grown in minimal essential medium (MEM) supplemented with 10% heat-inactivated fetal bovine serum (HI-FBS; Atlanta Biologicals), 0.1 mM nonessential amino acids, 1 mM sodium pyruvate, 100 U/ml penicillin, and 100 μg/ml streptomycin (Invitrogen). Huh7 cells were grown in Dulbecco's modified Eagle's medium (DMEM) supplemented with 10% HI-FBS, 100 U/ml penicillin, and 100 μg/ml streptomycin. The human choriocarcinoma cell line BeWo (ATCC CCL-98) was a gift from John Mitchell Robinson (The Ohio State University, OH, USA). BeWo cells were grown in DMEM-F12 medium (1:1) supplemented with 10% HI-FBS, 100 U/ml penicillin, and 100 μg/ml streptomycin. Human umbilical vein endothelial cells (HUVECs; ScienCell Research Laboratories, San Diego, CA, USA) were cultured in endothelial cell medium (ECM) with 5% HI-FBS, endothelial cell growth supplement (ECGS; ScienCell), 100 U/ml penicillin, and 100 μg/ml streptomycin. All plates and flasks used for HUVEC culture were coated with 2 μg/cm^2^ human fibronectin (BD Biosciences).

### Western blotting (LLO, InlB, c-Met).

Bacterial lysates were loaded at several dilutions (8 × 10^7^, 4 × 10^7^, and 2 × 10^7^ bacteria loaded for LLO, and 3.2 × 10^8^, 1.6 × 10^8^, and 8 × 10^7^ bacteria loaded for InlB) and subjected to SDS-PAGE and Western blot analysis using polyvinylidene difluoride (PVDF) membranes and anti-LLO antibody (rabbit polyclonal from Abcam), anti-InlB antibody (rabbit polyclonal from Genscript), and secondary anti-rabbit IgG antibody conjugated to horseradish peroxidase (Cell Signaling). For detection of InlB production in prfA* mutants, 1.6 × 10^8^ cells were used. We also probed for p60 as a loading control (Adipogen). Signal detection was performed using an Amersham ECL select reagent kit (GE Healthcare) and a ChemiDoc XRS imaging system (Bio-Rad). Densitometry analysis was performed by enclosing each protein band within a region of standard size, and the intensity of each band was measured using ImageJ gel analysis. Results were the average intensities calculated from three independent experiments. All intensities were set relative to that of WT bacterial lysates. For detection of c-MET, hepatocytes were grown to 80% confluence under the same experimental conditions as those used for invasion assays. Cell lysates were subjected to SDS-PAGE and Western blot analysis using PVDF membranes with anti-c-MET (4F8.2; Millipore) antibodies and secondary anti-mouse IgG antibodies conjugated to horseradish peroxidase (Cell Signaling). Signal detection was performed as described above.

### Measuring bacterial association and internalization.

HepG2 (10^5^ cells/well), Hep3B (0.75 × 10^5^ cells/well), PLC5 (0.75 × 10^5^ cells/well), Huh7 (0.75 × 10^5^ cells/well), and HUVECs (2 × 10^4^ cells/well) were cultured in 24-well tissue culture plates on glass coverslips at 37°C in a 5% CO_2_ atmosphere for 48 h before infection. BeWo cells (0.85 × 10^4^ cells/well) were cultured in 24-well tissue culture plates on glass coverslips coated in 0.2% gelatin for 72 h before infection. The hepatocyte cell lines were infected with L. monocytogenes at an MOI of 20 and HUVECs at an MOI of 5; BeWo cells were infected with 10^6^ bacteria/well. Infection of hepatocytes with prfA* bacterial strains was performed at an MOI of 5 to avoid toxicity of LLO in prfA* strains. Plates were centrifuged for 5 min (500 × *g*) at room temperature and incubated for 30 min at 37°C. Cells were washed three times with PBS, fixed with 4% paraformaldehyde (PFA) in PBS for 15 min at room temperature, and blocked for 1 h in 0.1 M glycine and 10% HI-FBS in PBS, pH 7.4. Extracellular bacteria were labeled with anti-L. monocytogenes rabbit polyclonal antibodies (GeneTex) and with anti-rabbit secondary antibodies conjugated to Alexa Fluor 488 (Molecular Probes). Samples were permeabilized with 0.5% Triton X-100, and total (extracellular and intracellular) bacteria were labeled with anti-L. monocytogenes antibodies and secondary antibodies conjugated to Alexa Fluor 568 (Molecular Probes). Slides were mounted in ProLong gold antifade mountant containing DAPI (4′,6-diamidino-2-phenylindole; Molecular Probes) to stain nuclei. To quantify the number of cells, images (phase contrast, DAPI, Alexa Fluor 488, and Alexa Fluor 568) were automatically acquired for each experimental condition using the 20× objective. MetaMorph analysis software was used to enumerate the total numbers of bacteria (*N_t_*), extracellular bacteria (*N_e_*), and mammalian cells (*N_c_*) (61). The efficiency of bacterial internalization was calculated as follows: internalization = [(*N_t_* – *N_e_*)/*N_t_*] × 100. The efficiency of bacterial association was calculated as follows: association = *N_t_*/*N_c_*. For each experimental condition, a minimum of 100 bacteria were counted (this applies to bacterial mutants with the lowest association efficiency) and a minimum of 150 mammalian cells (this applies to Hep3B, which are the largest cells and the cells with which L. monocytogenes associates the most effectively). The average numbers of WT bacteria and corresponding mammalian cells counted in each experiment are indicated in the figure legends. Because BeWo cells clustered in a fashion that made individual cell nuclei challenging to enumerate, we quantified the cell surface area by tracing plasma membrane outlines in MetaMorph and determined the surface area in μm^2^. We then calculated the efficiency of bacterial association as follows: association = *N_t_/*cell surface area (μm^2^).

### Polystyrene bead coating with recombinant InlB and invasion assay.

Blue fluorescent carboxylate-modified latex beads (1-μm diameter; Molecular Probes) were coated covalently with a mixture of recombinant InlB (5 mg/ml) and BSA (5 mg/ml) according to the manufacturer's instructions. Control, BSA-coated beads were prepared with 10 mg/ml BSA under the same conditions. The beads were then washed three times with 0.33× PBS, pH 7.4, and stored at 4°C. To assess the capacity for InlB-coated beads to be ingested by hepatocytes, HepG2 cells were seeded in 24-well plates on cover glasses for 48 h, as described for bacterial invasion assays. Cells were washed with MEM, and InlB/BSA- or BSA-coated beads were added to the wells at an MOI of 5. Plates were centrifuged for 3 min at 500 × *g* and incubated for 30 min at 37°C in 5% CO_2_. Cells were washed with PBS, fixed with 4% paraformaldehyde (PFA) in PBS for 15 min at room temperature, and washed and blocked for 1 h in 0.1 M glycine and 5% blotting-grade blocker (Bio-Rad) in PBS, pH 7.4. Extracellular beads were labeled with rabbit anti-BSA antibodies (Sigma-Aldrich; B1520), followed by anti-rabbit secondary antibodies conjugated to Alexa Fluor 488. Slides were mounted in ProLong gold antifade mountant containing DAPI to stain the nuclei. The percentage of intracellular beads was determined by fluorescence microscopy. The percentage of intracellular beads was calculated as the number of intracellular beads divided by the total number of beads, multiplied by 100.

### Live-cell imaging to assess hepatocyte response to InlB.

Hepatocytes were seeded (HepG2, 4 × 10^5^ cells/dish; Hep3B, PLC5, and Huh7, 3 × 10^5^ cells/dish) in 35-mm-diameter imaging dishes (Matek; P35G-1.5-10-C) and cultured at 37°C in 5% CO_2_ for 48 h. Cells were placed on the 37°C microscope stage and incubated with cell imaging medium without phenol red. Differential interference contrast (DIC) images were acquired with the 63× objective every 20 s for 15 min. At 5 min after the start of imaging, recombinant InlB was added to the cell culture medium to a final concentration of 1 nM. Under the control condition, the cells were imaged for 15 min without InlB.

### Western blotting of Akt phosphorylation.

Hepatocytes were seeded (HepG2, 5 × 10^5^ cells/dish; Hep3B, PLC5, and Huh7, 3 × 10^5^ cells/dish) in 35-mm-diameter cell culture dishes and cultured for 48 h. For exposure to recombinant InlB, cells were washed and incubated for 30 min in serum-free medium and then incubated with or without 1.25 nM InlB for 5 min at 37°C. The cells were then washed with cold PBS and lysed with cold lysis buffer (150 mM NaCl, 20 mM Tris/HCl, 2 mM EDTA, 1% NP-40, 3 mM sodium orthovanadate, 50 mM sodium fluoride, and 1× EDTA-free protease inhibitor cocktail [Roche]). To assess the effect of InlB produced by L. monocytogenes, the cells were washed with medium without serum and infected with WT or InlB-deficient bacteria at an MOI of 20 for 30 min at 37°C (same experimental conditions as the invasion assay). The cells were then washed and lysed. Cell lysates were subjected to Western blot analysis using PVDF membranes and anti-Akt or anti-phospho-Akt (Ser473) antibodies (Cell Signaling) and secondary anti-rabbit IgG antibodies conjugated to horseradish peroxidase (Cell Signaling).

### Microscope equipment.

Images were acquired on a motorized, inverted, wide-field fluorescence microscope (Axio Observer D1, TempModule S, heating unit XL S; Zeiss) equipped with a PZ-2000 XYZ automated stage, 20× Plan Neofluar (numerical aperture [NA] = 0.5), 40× Plan Neofluar (NA = 1.3), and 63× Plan Apochromat (NA = 1.4) objectives, a high-speed Xenon fluorescence emission device (Lambda DG-4, 300 W; Sutter Instrument Company), a Lambda 10-3 optical emission filter wheel for the fluorescence imaging, a SmartShutter to control the illumination for phase-contrast and DIC imaging (Sutter Instrument Company), a back-illuminated, frame-transfer electron-multiplying charge-coupled device (EMCCD) camera (Cascade II 512; Photometrics), and an ORCA-Flash 4.0 sCMOS camera (Hamamatsu). The filter sets for fluorescence were purchased from Chroma Technology Corporation and were as follows: DAPI (49000), Alexa Fluor 488 (49002), Alexa Fluor 568 (49005), and Cy5 (49006). Images were acquired and analyzed using MetaMorph imaging software (Molecular Devices).

### Statistical methods.

All experimental work involved at least three biological replicates, each performed on different days. Data obtained each day include different treatment conditions, which are considered a cluster. Data within the same cluster are more correlated to each other than to data from clusters obtained on different days. Linear mixed-effects models were used to account for the correlation among observations from a same cluster. Linear mixed-effects models were used to analyze data from invasion assays (bacterial entry and association), studies of the interaction between invasion proteins, RT-qPCR, and quantitative Western blot analyses. For RT-qPCR and Western blot analyses, data were first normalized to internal controls or the loading standard to reduce variation before analysis. Holm's procedure was used to adjust for multiple comparisons such as comparisons of each L. monocytogenes deletion mutant to the WT. SAS 9.4 was used for all analyses (SAS Institute, Inc., NC). Although normalized data were presented in some figures for a clearer visualization of results, all statistical analyses were performed on raw data before normalization.

## Supplementary Material

Supplemental file 1

Supplemental file 2

Supplemental file 3

Supplemental file 4

Supplemental file 5
