# Supplementary material for: Relative Roles of Listeriolysin O, InlA, and InlB in Listeria monocytogenes Uptake by Host Cells
Source: Infect Immun. 2018 Sep 21;86(10):e00555-18. doi: 10.1128/IAI.00555-18 (PMC6204736; doi:10.1128/IAI.00555-18)
Supplement: Supplemental file 1 [file zii999092560s1.pdf]

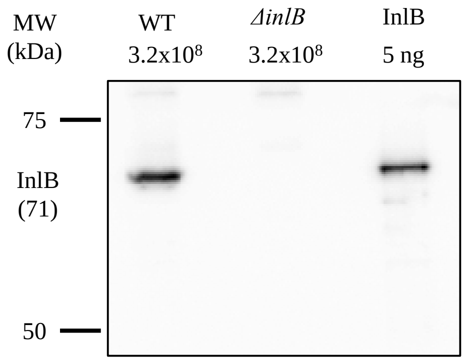

Supplemental Figure 1. Validation of anti-InlB antibodies.
Cell lysates of WT and InlB-deficient (Δ*inlB*) *L. monocytogenes* ($3.2x10^8$ bacteria), and 5 ng of InlB (used as a positive control) were subjected to western blot analysis using the anti-InlB polyclonal rabbit antibodies.

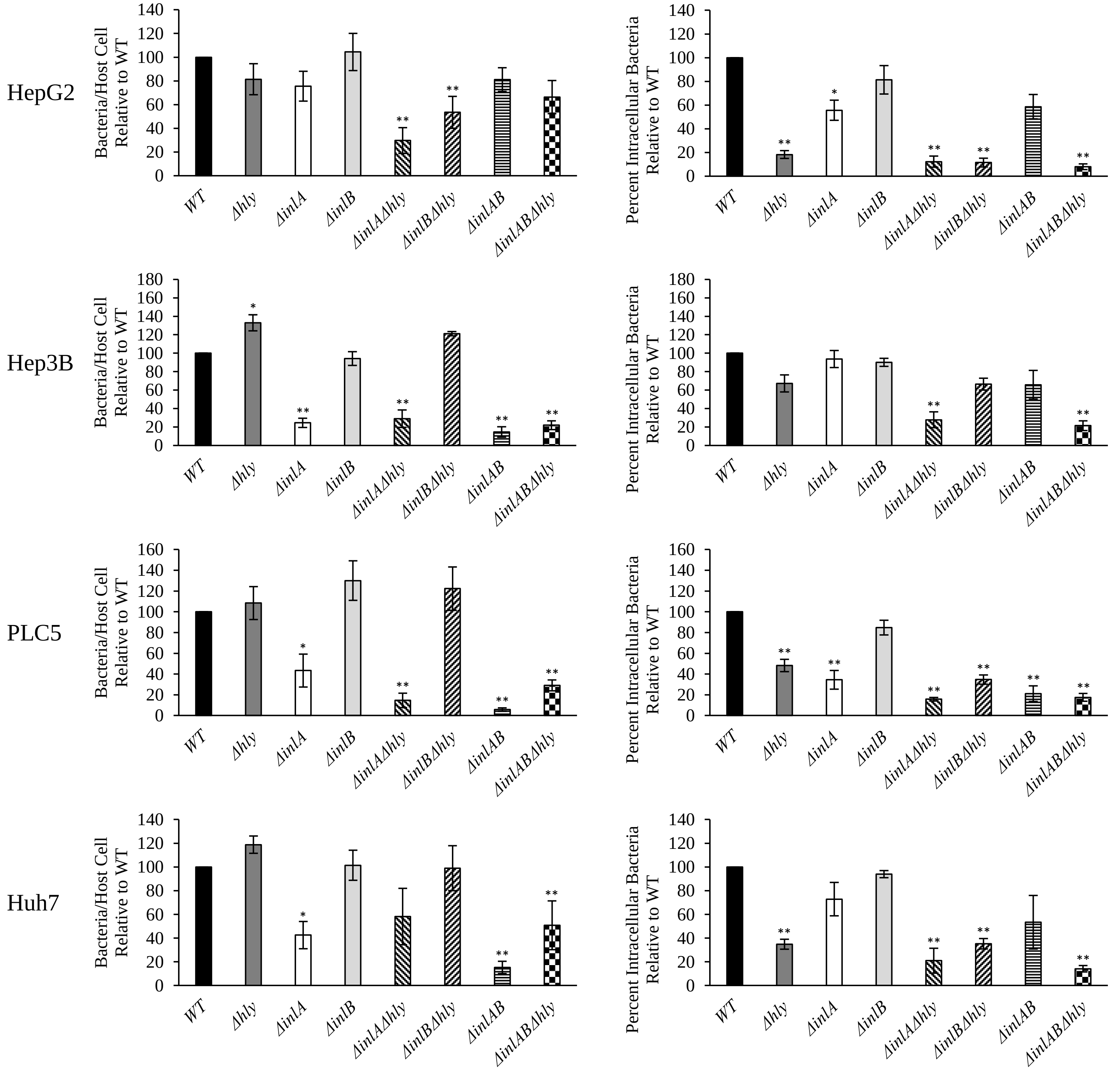

Supplemental Figure 2. Cooperation of LLO, InlA, and InlB in bacterial invasion of human hepatocytes. Hepatocyte cell lines were infected with WT, LLO-deficient (*Δhly*), InlA-deficient (*ΔinlA*), InlB-deficient (*ΔinlB*), InlA/LLO-deficient (*ΔinlAΔhly*), InlB/LLO-deficient (*ΔinlBΔhly*), InlAB-deficient (*ΔinlAB*), and InlAB/LLO-deficient (*ΔinlABΔhly*) bacteria (MOI 20) for 30 min at 37°C. Cells were washed, fixed and labeled with fluorescent antibodies and DAPI. (Left Column) Total bacteria/host cell. The average bacterial association for the WT strain before normalization were: HepG2 – 0.14, Hep3B – 3.13, PLC5 – 1.34, Huh7 – 0.77. (Right Column) Percent intracellular bacteria. The average internalization values for the WT strain before normalization were: HepG2 – 26.45%, Hep3B – 38.77%, PLC5 – 18.29%, Huh7 – 33.12 %. The minimum number of host cells counted was: HepG2 – 1000, Hep3B – 150, PLC5 – 600, Huh7 – 2000. The average number of WT bacteria counted per experiment was: HepG2 – 600, Hep3B – 4,000, PLC5 – 2,000, Huh7 – 3,000 with a minimum count of 100 bacteria being required for any mutant with reduced association efficiency. Results are expressed as the mean ± SEM relative to WT ($n \geq 3$). Statistical analyses compared each strain to the WT strain and were performed on raw data before normalization (* $p < 0.01$, ** $p < 0.001$).

In the following movies, images were acquired with a 6X DIC objective every 20 s for a total of 920 s.

Movie S1. Phase contract movie of HepG2 cells incubated at 37°C and exposed to 1 nM rInlB after 5 min. Arrow indicates membrane ruffles. Scale bar is 10 µm.

Movie S2. Phase contract movie of Hep3B cells incubated at 37°C and exposed to 1 nM rInlB after 5 min. Arrow indicates membrane ruffles. Scale bar is 10 µm.

Movie S3. Phase contract movie of PLC5 cells incubated at 37°C and exposed to 1 nM rInlB after 5 min. Arrows indicate membrane ruffles. Scale bar is 10 µm.

Movie S4. Phase contract movie of Huh7 cells incubated at 37°C and exposed to 1 nM rInlB after 5 min. Arrows indicate membrane ruffles. Scale bar is 10 µm.
